# Supplementary material for: Deleterious Effects of Yoyo Dieting and Resistant Starch on Gastrointestinal Morphology
Source: Nutrients. 2024 Dec 6;16(23):4216. doi: 10.3390/nu16234216 (PMC11644255; doi:10.3390/nu16234216)
Supplement: Supplementary file 1 [file nutrients-16-04216-s001.zip › Supplementary/sf17-091_Control.pdf]

3150 Great Eastern Hwy
Glen Forrest
Western Australia 6071
p: +61 8 9298 8111
F: +61 8 9298 8700
Email: info@specialtyfeeds.com

Diet
SF17-091 Low Fat Rodent Diet Based on D12450B For Irradiation

A semi-pure high fat diet formulation for laboratory rats and mice based on Research Diets D12450B. Some modifications have been made to the original formulation to suit locally available raw materials.

- Vitamin levels have been increased for irradiation

Calculated Nutritional Parameters	
Protein	23.0%
Total Fat	5.3%
Crude Fibre	5.4%
AD Fibre	5.4%
Digestible Energy	15.6 MJ / Kg
% Total calculated digestible energy from lipids	12.3%
% Total calculated digestible energy from protein	25.5%

Diet Form and Features

- Semi pure low fat diet. 12 mm diameter pellets.
- Pack size 5 Kg , vacuum packed in oxygen impermeable plastic bags, under nitrogen. Bags are packed into cardboard cartons to protect them during transit. Smaller pack quantity on request.
- Diet suitable for irradiation but not suitable for autoclave.
- Lead time 2 weeks for non-irradiation or 4 weeks for irradiation.

Ingredients	
Casein (Acid)	233 g/Kg
Sucrose	201 g/Kg
Lard	23 g/Kg
Soya Bean Oil	29 g/Kg
Cellulose	58 g/Kg
Wheat Starch	270 g/Kg
Dextrinised Starch	117 g/Kg
L Methionine	3.5 g/Kg
Calcium Carbonate	6.4 g/Kg
Sodium Chloride	2.6 g/Kg
AIN93 Trace Minerals	1.6 g/Kg
Potassium Citrate	19.2 g/Kg
Dicalcium Phosphate	15.1 g/Kg
Potassium Sulphate	1.6 g/Kg
Choline Chloride (75%)	1.3 g/Kg
AIN93 Vitamins	16.5 g/Kg
Vitamin K 0.23%	0.87 g/Kg

Calculated Amino Acids as Fed

Valine	1.46%
Leucine	1.99%
Isoleucine	1.10%
Threonine	0.92%
Methionine	0.97%
Cysteine	0.09%
Lysine	1.65%
Phenylalanine	1.09%
Tyrosine	1.15%
Tryptophan	0.20%
Histidine	0.61%

Calculated Total Minerals as Fed

Calcium	0.69%
Phosphorous	0.48%
Magnesium	0.08%
Sodium	0.14%
Chloride	0.16%
Potassium	0.83%
Sulphur	0.26%
Iron	70 mg/Kg
Copper	9.4 mg/Kg
Iodine	0.2 mg/Kg
Manganese	22 mg/Kg
Cobalt	No data
Zinc	55 mg/Kg
Molybdenum	0.17 mg/Kg
Selenium	0.4 mg/Kg
Cadmium	No data
Chromium	1.2 mg/Kg
Fluoride	1.2 mg/Kg
Lithium	0.1 mg/Kg
Boron	1.8 mg/Kg
Nickel	0.6 mg/Kg
Vanadium	0.1 mg/Kg

Calculated Total Vitamins as Fed

Vitamin A (Retinol)	6 660 IU/Kg
Vitamin D (Cholecalciferol)	1 650 IU/Kg
Vitamin E (a Tocopherol acetate)	126 mg/Kg
Vitamin K (Menadione)	3.7 mg/Kg
Vitamin C (Ascorbic acid)	None added
Vitamin B1 (Thiamine)	10.1 mg/Kg
Vitamin B2 (Riboflavin)	10.3 mg/Kg
Niacin (Nicotinic acid)	50 mg/Kg
Vitamin B6 (Pryridoxine)	12 mg/Kg
Pantothenic Acid	27 mg/Kg
Biotin	333 ug/Kg
Folic Acid	3.4 mg/Kg
Inositol	None added
Vitamin B12 (Cyancobalamin)	170 ug/Kg
Choline	1 210 mg/Kg

Calculated Fatty Acid Composition as Fed

Saturated Fats C12:0 or less	Trace
Myristic Acid 14:0	0.04%
Palmitic Acid 16:0	0.92%
Stearic Acid 18:0	0.51%
Palmitoleic Acid 16:1	0.05%
Oleic Acid 18:1	1.45%
Gadoleic Acid 20:1	0.02%
Linoleic Acid 18:2 n6	1.82%
a Linolenic Acid 18:3 n3	0.23%
EPA 20:5 n3	No data
DHA 22:6 n3	No data
Total n3	0.23%
Total n6	1.82%
Total Mono Unsaturated Fats	1.53%
Total Polyunsaturated Fats	2.07%
Total Saturated Fats	1.50%

Calculated data uses information from typical raw material composition. It could be expected that individual batches of diet will vary from this figure. **Diet post treatment by irradiation or autoclave could change these parameters**.
We are happy to provide full calculated nutritional information for all of our products, however we would like to emphasise that these diets have been specifically designed for manufacture by Specialty Feeds.
