# Supplementary material for: Deleterious Effects of Yoyo Dieting and Resistant Starch on Gastrointestinal Morphology
Source: Nutrients. 2024 Dec 6;16(23):4216. doi: 10.3390/nu16234216 (PMC11644255; doi:10.3390/nu16234216)
Supplement: Supplementary file 1 [file nutrients-16-04216-s001.zip › Supplementary/sf21-019_Control with RS.pdf]

3150 Great Eastern Hwy
Glen Forrest
Western Australia 6071
p: +61 8 9298 8111
F: +61 8 9298 8700
Email: info@specialtyfeeds.com

Specialty Feeds

Diet

SF21-019

Resistant Starch Modified SF13-081

A semi-pure low fat diet formulation for laboratory rats and mice based on SF13-081.

- Dextrinised starch has been replaced with a high resistant starch Gemstar RS.
- Fibre compont of Gemstar RS is assumed to be crude fibre.
- No other changes have been made to the formulation
- Diet is control for SF21-018
- Vitamins have been increased for irradiation
- See data sheet SF13-081 for more information on the base diet.

Calculated Nutritional Parameters

Protein	23.0%
Total Fat	5.3%
Crude Fibre	15.3%
AD Fibre	15.3%
Digestible Energy	13.6 MJ / Kg
% Total calculated digestible energy from lipids	13.9%
% Total calculated digestible energy from protein	27.8%

Diet Form and Features

- Semi pure low fat diet. 12 mm diameter pellets.
- Pack size 5 Kg , vacuum packed in oxygen impermeable plastic bags, under nitrogen. Bags are packed into cardboard cartons to protect them during transit. Smaller pack quantity on request.
- Diet suitable for irradiation but not suitable for autoclave.
- Lead time 2 weeks for non-irradiation or 4 weeks for irradiation.

Ingredients

Casein (Acid)	233 g/Kg
Sucrose	201 g/Kg
Lard	23 g/Kg
Soya Bean Oil	29 g/Kg
Cellulose	58 g/Kg
Wheat Starch	275 g/Kg
GemStar RS Starch	117 g/Kg
L Methionine	3.5 g/Kg
Calcium Carbonate	6.4 g/Kg
Sodium Chloride	2.6 g/Kg
AIN93 Trace Minerals	1.6 g/Kg
Potassium Citrate	19.2 g/Kg
Dicalcium Phosphate	15.1 g/Kg
Potassium Sulphate	1.6 g/Kg
Choline Chloride (75%)	1.3 g/Kg
AIN93 Vitamins	16.5 g/Kg
Vitamin K 0.23%	0.87 g/Kg

FEEDSAFE ACCREDITED FEED MANUFACTURER
JAS-ANZ
ISO 9001
SGS
AUSTRALIAN TRUSTED TRADER
INTERNATIONAL AEO AND TRADE FACILITATION

Calculated Amino Acids as Fed	
Valine	1.19%
Leucine	1.63%
Isoleucine	0.90%
Threonine	0.75%
Methionine	0.79%
Cysteine	0.07%
Lysine	1.35%
Phenylalanine	0.89%
Tyrosine	0.94%
Tryptophan	0.16%
Histidine	0.50%

Calculated Total Minerals as Fed	
Calcium	0.69%
Phosphorous	0.48%
Magnesium	0.08%
Sodium	0.14%
Chloride	0.16%
Potassium	0.83%
Sulphur	0.26%
Iron	70 mg/Kg
Copper	9.4 mg/Kg
Iodine	0.2 mg/Kg
Manganese	22 mg/Kg
Cobalt	No data
Zinc	55 mg/Kg
Molybdenum	0.17 mg/Kg
Selenium	0.4 mg/Kg
Cadmium	No data
Chromium	1.2 mg/Kg
Fluoride	1.2 mg/Kg
Lithium	0.1 mg/Kg
Boron	1.8 mg/Kg
Nickel	0.6 mg/Kg
Vanadium	0.1 mg/Kg

Calculated Total Vitamins as Fed	
Vitamin A (Retinol)	6 660 IU/Kg
Vitamin D (Cholecalciferol)	1 670 IU/Kg
Vitamin E (a Tocopherol acetate)	126 mg/Kg
Vitamin K (Menadione)	3.7 mg/Kg
Vitamin C (Ascorbic acid)	None added
Vitamin B1 (Thiamine)	10.1 mg/Kg
Vitamin B2 (Riboflavin)	10.3 mg/Kg
Niacin (Nicotinic acid)	50 mg/Kg
Vitamin B6 (Pryridoxine)	12 mg/Kg
Pantothenic Acid	27 mg/Kg
Biotin	333 ug/Kg
Folic Acid	3.4 mg/Kg
Inositol	None added
Vitamin B12 (Cyancobalamin)	170 ug/Kg
Choline	1 190 mg/Kg

Calculated Fatty Acid Composition as fed	
Saturated Fats C12:0 or less	Trace
Myristic Acid 14:0	0.04%
Palmitic Acid 16:0	0.92%
Stearic Acid 18:0	0.51%
Palmitoleic Acid 16:1	0.05%
Oleic Acid 18:1	1.45%
Gadoleic Acid 20:1	0.02%
Linoleic Acid 18:2 n6	1.82%
a Linolenic Acid 18:3 n3	0.23%
EPA 20:5 n3	No data
DHA 22:6 n3	No data
Total n3	0.23%
Total n6	1.82%
Total Mono Unsaturated Fats	1.53%
Total Polyunsaturated Fats	2.07%
Total Saturated Fats	1.50%

Calculated data uses information from typical raw material composition. It could be expected that individual batches of diet will vary from this figure. **Diet post treatment by irradiation or autoclave could change these parameters**.
We are happy to provide full calculated nutritional information for all of our products, however we would like to emphasise that these diets have been specifically designed for manufacture by Specialty Feeds.
