# Supplementary material for: Deleterious Effects of Yoyo Dieting and Resistant Starch on Gastrointestinal Morphology
Source: Nutrients. 2024 Dec 6;16(23):4216. doi: 10.3390/nu16234216 (PMC11644255; doi:10.3390/nu16234216)
Supplement: Supplementary file 1 [file nutrients-16-04216-s001.zip › Supplementary/sf21-018_High fat with RS.pdf]

3150 Great Eastern Hwy
Glen Forrest
Western Australia 6071
p: +61 8 9298 8111
F: +61 8 9298 8700
Email: info@specialtyfeeds.com

Diet SF21-018 — Resistant Starch Modification of SF13-092 P20 F60 C20

A semi-pure high fat diet formulation for laboratory rats and mice based on SF13-092.

- Dextrinised starch is replace with GEMSTAR RS, a very high resistant wheat starch at 140 g/Kg wheat starch has been added.
- The fibre component of Gemstar RS is assumed to be crude fibre.
- Casein and Lard inclusion has reduced to keep the energy proportion constant.
- Vitamins have been increased for irradiation
- See Below for energy brake down.

Calculated Nutritional Parameters

Protein	22.0%
Total Fat	29.6%
Crude Fibre	17.9%
AD Fibre	17.9%
Digestible Energy	18.1 MJ / Kg
% Total calculated digestible energy from lipids	60.0%
% Total calculated digestible energy from protein	20.0%

Diet Form and Features

- Semi pure high fat diet. 12 mm diameter pellets.
- Pack size 1.5 Kg trays, vacuum packed in oxygen impermeable plastic bags, under nitrogen. Bags are packed into cardboard cartons to protect them during transit. Smaller pack quantity on request.
- Diet suitable for irradiation but not suitable for autoclave.
- Lead time 2 weeks for non-irradiation or 4 weeks for irradiation.
- Diet is light blue in colour

Ingredients

Casein (Acid)	222 g/Kg
Sucrose	88.9 g/Kg
Lard	265 g/Kg
Soya Bean Oil	32.3 g/Kg
Cellulose	64.6 g/Kg
Wheat Starch	110 g/Kg
Gemstar RS	140 g/Kg
L Methionine	3.9 g/Kg
Calcium Carbonate	7.1 g/Kg
Sodium Chloride	2.9 g/Kg
AIN93 Trace Minerals	1.8 g/Kg
Potassium Citrate	21.3 g/Kg
Dicalcium Phosphate	16.8 g/Kg
Potassium Sulphate	1.8 g/Kg
Choline Chloride (75%)	2.6 g/Kg
AIN93 Vitamins	17.9 g/Kg
Vitamin K 0.23%	0.87 g/Kg

Calculated Essential Amino Acids as Fed	
Valine	1.47%
Leucine	2.09%
Isoleucine	1.18%
Threonine	0.95%
Methionine	1.08%
Cysteine	0.14%
Lysine	1.82%
Phenylalanine	1.15%
Tyrosine	1.31%
Tryptophan	0.29%
Arginine	0.87%
Histidine	0.62%

Calculated Total Minerals as Fed	
Calcium	0.75%
Phosphorous	0.48%
Magnesium	0.08%
Sodium	0.14%
Chloride	0.18%
Potassium	0.91%
Sulphur	0.26%
Iron	77 mg/Kg
Copper	10 mg/Kg
Iodine	0.26 mg/Kg
Manganese	21 mg/Kg
Cobalt	No data
Zinc	59 mg/Kg
Molybdenum	0.2 mg/Kg
Selenium	0.3 mg/Kg
Cadmium	No data
Chromium	1.3 mg/Kg
Fluoride	1.3 mg/Kg
Lithium	0.1 mg/Kg
Boron	1.7 mg/Kg
Nickel	0.6 mg/Kg
Vanadium	0.1 mg/Kg

Calculated Total Vitamins as Fed	
Vitamin A (Retinol)	7 160 IU/Kg
Vitamin D (Cholecalciferol)	1 790 IU/Kg
Vitamin E (a Tocopherol acetate)	136 mg/Kg
Vitamin K (Menadione)	3.8 mg/Kg
Vitamin C (Ascorbic acid)	None added
Vitamin B1 (Thiamine)	10.8 mg/Kg
Vitamin B2 (Riboflavin)	11 mg/Kg
Niacin (Nicotinic acid)	54 mg/Kg
Vitamin B6 (Pryridoxine)	13 mg/Kg
Pantothenic Acid	29 mg/Kg
Biotin	358 ug/Kg
Folic Acid	3.6 mg/Kg
Inositol	None added
Vitamin B12 (Cyancobalamin)	180 ug/Kg
Choline	2 220 mg/Kg

Calculated Fatty Acid Composition as Fed	
Saturated Fats C12:0 or less	0.08%
Myristic Acid 14:0	0.40%
Palmitic Acid 16:0	7.34%
Stearic Acid 18:0	4.68%
Other Saturated Fats	0.22%
Palmitoleic Acid 16:1	0.46%
Oleic Acid 18:1	9.68%
Gadoleic Acid 20:1	0.20%
Linoleic Acid 18:2 n6	5.46%
a Linolenic Acid 18:3 n3	0.59%
EPA 20:5 n3	No data
DHA 22:6 n3	No data
Total n3	0.63%
Total n6	5.49%
Total Mono Unsaturated Fats	10.42%
Total Poly Unsaturated Fats	6.26%
Total Saturated Fats	12.75%

Calculated data uses information from typical raw material composition. It could be expected that individual batches of diet will vary from this figure. **Diet post treatment by irradiation or autoclave could change these parameters**. We are happy to provide full calculated nutritional information for all of our products, however we would like to emphasise that these diets have been specifically designed for manufacture by Specialty Feeds.
