# Supplementary material for: Deleterious Effects of Yoyo Dieting and Resistant Starch on Gastrointestinal Morphology
Source: Nutrients. 2024 Dec 6;16(23):4216. doi: 10.3390/nu16234216 (PMC11644255; doi:10.3390/nu16234216)
Supplement: Supplementary file 1 [file nutrients-16-04216-s001.zip › Supplementary/sf16-048_High fat.pdf]

Specialty Feeds
3150 Great Eastern Hwy
Glen Forrest
Western Australia 6071
p: +61 8 9298 8111
F: +61 8 9298 8700
Email: info@specialtyfeeds.com

Diet

SF16-048

High Fat Rodent Diet Based on D12492 Extra Vitamins For Irradiation

A semi-pure very high fat diet formulation for laboratory rats and mice based on Research Diets D12492. Some modifications have been made to the original formulation to suit locally available raw materials.

- We have evidence that vitamin losses and other changes to the diet can occur during irradiation at 25KGy. Vitamin levels have been increased to account for the expected losses.

Calculated Nutritional Parameters

Protein	25.50%
Total Fat	34.50%
Crude Fibre	6.00%
AD Fibre	6.00%
Digestible Energy	21.5 MJ / Kg
% Total calculated digestible energy from lipids	60.10%
% Total calculated digestible energy from protein	20.40%

Diet Form and Features

- Semi pure diet. 15mm x 20mm block to mimic similar size of pellet.
- Packed in plastic trays. Trays packed in groups of five (5). with layer of glad wrap between each to protect diet.
- Vacuum packed under nitrogen in oxygen impermeable bags. Packed in cardboard cartons for protection during transit.
- Diet must be stored at or below 15ºC
- Diet suitable for irradiation but not suitable for autoclave.
- Lead time 2 weeks for non-irradiation or 4 weeks for irradiation.
- Diet is light blue in colour

Ingredients

Casein (Acid)	258 g/Kg
Sucrose	88.9 g/Kg
Lard	317 g/Kg
Soya Bean Oil	32.3 g/Kg
Cellulose	64.6 g/Kg
Dextrinised Starch	162 g/Kg
L Methionine	3.9 g/Kg
Calcium Carbonate	7.1 g/Kg
Sodium Chloride	2.9 g/Kg
AIN93 Trace Minerals	1.8 g/Kg
Potassium Citrate	21.3 g/Kg
Dicalcium Phosphate	16.8 g/Kg
Potassium Sulphate	1.8 g/Kg
Choline Chloride (75%)	2.6 g/Kg
AIN93 Vitamins	17.9 g/Kg
Vitamin K 0.23%	0.87 g/Kg

FEEDSAFE ACCREDITED FEED MANUFACTURER
JAS-ANZ
ISO 9001 SGS
AUSTRALIAN TRUSTED TRADER
INTERNATIONAL AEO AND TRADE FACILITATION

Calculated Essential Amino Acids as Fed	
Valine	1.63%
Leucine	2.33%
Isoleucine	1.13%
Threonine	1.03%
Methionine	1.08%
Cysteine	0.08%
Lysine	1.93%
Phenylanine	1.28%
Tyrosine	1.35%
Tryptophan	0.35%
Histidine	0.78%

Calculated Total Minerals as Fed	
Calcium	0.79%
Phosphorous	0.51%
Magnesium	0.08%
Sodium	0.15%
Chloride	0.18%
Potassium	0.91%
Sulphur	0.28%
Iron	72 mg/Kg
Copper	10 mg/Kg
Iodine	0.26 mg/Kg
Manganese	22 mg/Kg
Cobalt	No data
Zinc	60 mg/Kg
Molybdenum	0.2 mg/Kg
Selenium	0.4 mg/Kg
Cadmium	No data
Chromium	1.3 mg/Kg
Fluoride	1.3 mg/Kg
Lithium	0.1 mg/Kg
Boron	2.5 mg/Kg
Nickel	0.6 mg/Kg
Vanadium	0.1 mg/Kg

Calculated Total Vitamins as Fed	
Vitamin A (Retinol)	7 160 IU/Kg
Vitamin D (Cholecalciferol)	1 790 IU/Kg
Vitamin E (a Tocopherol acetate)	136 mg/Kg
Vitamin K (Menadione)	3.8 mg/Kg
Vitamin C (Ascorbic acid)	None added
Vitamin B1 (Thiamine)	10.8 mg/Kg
Vitamin B2 (Riboflavin)	11.1 mg/Kg
Niacin (Nicotinic acid)	54 mg/Kg
Vitamin B6 (Pryridoxine)	12.8 mg/Kg
Pantothenic Acid	29 mg/Kg
Biotin	358 ug/Kg
Folic Acid	3.6 mg/Kg
Inositol	None added
Vitamin B12 (Cyancobalamin)	180 ug/Kg
Choline	2 090 mg/Kg

Calculated Fatty Acid Composition as Fed	
Saturated Fats C12:0 or less	0.10%
Myristic Acid 14:0	0.48%
Palmitic Acid 16:0	8.72%
Stearic Acid 18:0	5.57%
Other Saturated Fats	0.30%
Palmitoleic Acid 16:1	0.55%
Oleic Acid 18:1	11.44%
Gadoleic Acid 20:1	0.24%
Linoleic Acid 18:2 n6	6.21%
a Linolenic Acid 18:3 n3	0.66%
EPA 20:5 n3	No data
DHA 22:6 n3	No data
Total n3	0.71%
Total n6	6.24%
Total Mono Unsaturated Fats	12.33%
Total Poly Unsaturated Fats	7.12%
Total Saturated Fats	15.18%

Calculated data uses information from typical raw material composition. It could be expected that individual batches of diet will vary from this figure. **Diet post treatment by irradiation or autoclave could change these parameters**. We are happy to provide full calculated nutritional information for all of our products, however we would like to emphasise that these diets have been specifically designed for manufacture by Specialty Feeds.
